# Prioritization of potential pharmacological targets for the development of anti-hepatocarcinoma drugs modulating the extrinsic apoptosis pathway: the reconstruction and analysis of associative gene networks help

**DOI:** 10.18699/VJGB-23-91

**Published:** 2023-12

**Authors:** P.S. Demenkov, E.A. Antropova, A.V. Adamovskaya, E.L. Mishchenko, T.M. Khlebodarova, T.V. Ivanisenko, N.V. Ivanisenko, A.S. Venzel, I.N. Lavrik, V.A. Ivanisenko

**Affiliations:** Institute of Cytology and Genetics of the Siberian Branch of the Russian Academy of Sciences, Novosibirsk, Russia Kurchatov Genomic Center of ICG SB RAS, Novosibirsk, Russia Novosibirsk State University, Novosibirsk, Russia; Institute of Cytology and Genetics of the Siberian Branch of the Russian Academy of Sciences, Novosibirsk, Russia; Institute of Cytology and Genetics of the Siberian Branch of the Russian Academy of Sciences, Novosibirsk, Russia Novosibirsk State University, Novosibirsk, Russia; Institute of Cytology and Genetics of the Siberian Branch of the Russian Academy of Sciences, Novosibirsk, Russia Kurchatov Genomic Center of ICG SB RAS, Novosibirsk, Russia; Institute of Cytology and Genetics of the Siberian Branch of the Russian Academy of Sciences, Novosibirsk, Russia Kurchatov Genomic Center of ICG SB RAS, Novosibirsk, Russia; Institute of Cytology and Genetics of the Siberian Branch of the Russian Academy of Sciences, Novosibirsk, Russia Kurchatov Genomic Center of ICG SB RAS, Novosibirsk, Russia Novosibirsk State University, Novosibirsk, Russia; Institute of Cytology and Genetics of the Siberian Branch of the Russian Academy of Sciences, Novosibirsk, Russia; Institute of Cytology and Genetics of the Siberian Branch of the Russian Academy of Sciences, Novosibirsk, Russia Kurchatov Genomic Center of ICG SB RAS, Novosibirsk, Russia Novosibirsk State University, Novosibirsk, Russia; Medical Faculty, Otto von Guericke University Magdeburg, Magdeburg, Germany; Institute of Cytology and Genetics of the Siberian Branch of the Russian Academy of Sciences, Novosibirsk, Russia Kurchatov Genomic Center of ICG SB RAS, Novosibirsk, Russia Novosibirsk State University, Novosibirsk, Russia

**Keywords:** gene networks, hepatocarcinoma, programmed cell death, apoptosis, methylation, генные сети, гепатокарцинома, программируемая клеточная гибель, апоптоз, метилирование

## Abstract

Hepatocellular carcinoma (HCC) is a common severe type of liver cancer characterized by an extremely aggressive course and low survival rates. It is known that disruptions in the regulation of apoptosis activation are some of the key features inherent in most cancer cells, which determines the pharmacological induction of apoptosis as an important strategy for cancer therapy. The computer design of chemical compounds capable of specifically regulating the external signaling pathway of apoptosis induction represents a promising approach for creating new effective ways of therapy for liver cancer and other oncological diseases. However, at present, most of the studies are devoted to pharmacological effects on the internal (mitochondrial) apoptosis pathway. In contrast, the external pathway induced via cell death receptors remains out of focus. Aberrant gene methylation, along with hepatitis C virus (HCV) infection, are important risk factors for the development of hepatocellular carcinoma. The reconstruction of gene networks describing the molecular mechanisms of interaction of aberrantly methylated genes with key participants of the extrinsic apoptosis pathway and their regulation by HCV proteins can provide important information when searching for pharmacological targets. In the present study, 13 criteria were proposed for prioritizing potential pharmacological targets for developing anti-hepatocarcinoma drugs modulating the extrinsic apoptosis pathway. The criteria are based on indicators of the structural and functional organization of reconstructed gene networks of hepatocarcinoma, the extrinsic apoptosis pathway, and regulatory pathways of virus-extrinsic apoptosis pathway interaction and aberrant gene methylation-extrinsic apoptosis pathway interaction using ANDSystem. The list of the top 100 gene targets ranked according to the prioritization rating was statistically significantly (p-value = 0.0002) enriched for known pharmacological targets approved by the FDA, indicating the correctness of the prioritization method. Among the promising potential pharmacological targets, six highly ranked genes (JUN, IL10, STAT3, MYC, TLR4, and KHDRBS1) are likely to deserve close attention.

## Abstract (ru)

Гепатоцеллюлярная карцинома (ГЦК) – распространенный тяжелый тип рака печени, характеризующийся крайне агрессивным течением и низкой выживаемостью. Известно, что нарушения регуляции активации апоптоза являются одной из ключевых особенностей, свойственной большинству раковых клеток, что определяет фармакологическую индукцию апоптоза как важную стратегию терапии рака. Компьютерный дизайн химических соединений, способных целевым образом регулировать внешний сигнальный путь индукции апоптоза, представляет перспективный подход для создания новых эффективных средств терапии рака печени и других онкологических заболеваний. Однако в настоящее время большинство исследований посвящено фармакологическим воздействиям на внутренний (митохондриальный) путь апоптоза, тогда как внешний путь, индуцируемый посредством клеточных рецепторов смерти, остается вне поля зрения. Аберрантное метилирование генов наряду с инфекцией вирусом гепатита С считаются важными факторами риска развития ГЦК. Реконструкция генных сетей, описывающих молекулярные механизмы взаимодействия аберрантно метилированных генов с ключевыми участниками внешнего пути апоптоза, а также пути их регуляции белками вируса гепатита С, может дать важную информацию при поиске фармакологических мишеней. В настоящей работе были предложены 13 критериев приоритизации потенциальных фармакологических мишеней для создания лекарств против гепатокарциномы, модулирующих внешний путь апоптоза. В основу критериев легли показатели структурно-функциональной организации реконструированных с использованием ANDSystem генных сетей ГЦК, внешнего пути апоптоза и регуляторных путей взаимодействия «вирус – внешний путь апоптоза» и «аберрантное метилирование генов – внешний путь апоптоза». Список наиболее приоритетных 100 генов-мишеней, ранжированных согласно рейтингу приоритизации, оказался статистически значимо ( p-value = 0.0002) обогащен известными фармакологическими мишенями, одобренными FDA, что указывает на корректность примененного метода приоритизации. Среди перспективных потенциальных фармакологических мишеней могут быть представлены шесть генов-кандидатов (JUN, IL10, STAT3, MYC, TLR4 и KHDRBS1), занимающих высокое положение в ранжированном списке согласно результатам приоритизации.

## Introduction

Hepatocellular carcinoma (HCC) is the most common tumor pathology of the liver, accounting for over 90 % of all malignant neoplasms of the liver and intrahepatic bile ducts (Llovet et al., 2018). It is characterized by an extremely aggressive course and low survival rate. Unlike most other types of cancer, there are some documented risk factors for the occurrence of HCC, such as infections caused by hepatitis C and B viruses, alcohol, fatty infiltration of the liver, hepatitis, autoimmune or chronic cholestatic diseases (Forner et al., 2012). Studies in the field of hepatocarcinogenesis have shown the critical role of genetic and epigenetic mechanisms leading to the formation of monoclonal populations of aberrant and dysplastic hepatocytes, which exhibit telomere erosion and re-expression of telomerase, microsatellite instability, and irreversible structural changes in genes and chromosomes (Balogh et al., 2016). The phenotype of malignant hepatocytes may be caused by the disruption of a number of genes that function in various regulatory pathways, resulting in different molecular variants of HCC (Thorgeirsson, Grisham, 2002). This characteristic of the pathology makes the reconstruction and analysis of gene networks describing the molecular mechanisms of the disease relevant.

In cancer therapeutic research, a central issue is suppressing cellular proliferation and the induction of programmed cell death. Apoptosis, one of the known mechanisms of programmed cell death, is divided into intrinsic and extrinsic, depending on the pathway of signal induction. The apoptosis signal induced by cell death receptors is called the extrinsic pathway, and the one induced by mitochondria – the intrinsic pathway (Krammer et al., 2007). In both cases, the apoptosis signal initiates the activation of caspases, key enzymes of apoptosis, leading to cell destruction, but the molecular mechanisms of signal transmission are entirely different. The literature focuses on regulating the intrinsic pathway of apoptosis, in which there has been certain progress in finding compounds with pharmacological potential for HCC therapy. It should be noted that the pharmacological effect on the extrinsic apoptosis pathway in HCC remains poorly studied. However, pharmacological induction of this pathway may bring significant, fundamentally important progress for cancer therapy.

Apoptosis induction is controlled by a range of inhibitor proteins, including c-FLIP, which blocks the activation of caspase-8, members of the anti-apoptotic BCl-2 family that inhibit the release of cytochromeC from mitochondria, and XIAP proteins that block the activation of caspase-3, -7, and -9. In the extrinsic apoptosis pathway, DISC, comprising PC, FADD, procaspase-8, -10 proteins, and c-FLIP, serves as a central platform for procaspase-8 activation (Lavrik, Krammer, 2012). c-FLIP can function within the DISC complex both pro- and anti-apoptotically. It is suggested that the formation of procaspase-8/c-FLIP heterodimers mediates the pro-apoptotic function of c-FLIP. Previously, in joint research conducted by the Institute of Cytology and Genetics of the Siberian Branch of the Russian Academy of Sciences and the University of Magdeburg, we developed the world’s first chemical probe (small chemical compound) capable of specifically binding to c-FLIP in the caspase-8/ c-FLIP heterodimeric complex (Hillert et al., 2020). This small molecule was obtained by computer design and possessed biological activity – the ability to increase caspase-8 activity (Hillert et al., 2020).

Hepatitis C virus (HCV) is extensively studied in the scientific literature as a significant risk factor for HCC (Axley et al., 2018). The role of HCV has been shown in the regulation of apoptosis and aberrant gene methylation, closely associated with HCC (Zheng et al., 2019; Lee, Ou, 2021).

Gene networks are widely used to describe the moleculargenetic mechanisms of various processes. We previously developed the software and information system ANDSystem (Ivanisenko V.A. et al., 2015, 2019; Ivanisenko T.V. et al., 2020, 2022), designed for the reconstruction and analysis of associative gene networks based on automatic knowledge extraction from scientific publications and factographic databases. Through the reconstruction of gene networks performed using ANDSystem, a number of studies have been conducted, such as the analysis of interactions of Hepatitis C virus proteins with the human proteome (Saik et al., 2016), the relationship of HCV with aberrant methylation in HCC (Antropova et al., 2022), interpretation of results of metabolome analysis of SARS-Cov-2 patients (Ivanisenko V.A. et al., 2022), tasks of prioritizing candidate genes associated with lymphedema, major depressive disorder (Yankina et al., 2018; Saik et al., 2019), search for new potential targets for drug action (Saik et al., 2018a, b), and others.

Based on the reconstruction and analysis of HCC gene networks and the extrinsic apoptosis pathway, as well as regulatory pathways linking HCV proteins with aberrantly methylated genes in HCC and key participants in the extrinsic apoptosis pathway, criteria were proposed for prioritizing potential pharmacological targets against HCC. Enrichment analysis of the first 100 target genes, ordered by prioritization results, showed significant content (p-value = 0.0002) in the list of FDA-approved pharmacological target genes, demonstrating the effectiveness of the proposed prioritization criteria. We suggest that the mechanism of action of drugs targeted at these targets is the modulation of the extrinsic apoptosis pathway, taking into account aberrant gene methylation, which could be utilized in creating a new class of drugs for HCC therapy. As promising potential pharmacological targets, ranked in the top thirty, the following candidate genes can be highlighted: JUN, IL10, STAT3, MYC, TLR4, and KHDRBS1.

## Materials and methods

The ANDSystem software and information tool. Gene network reconstruction was performed using the ANDSystem software and information tool, automatically extracting knowledge from scientific publications and factual databases using artificial intelligence methods (Ivanisenko V.A. et al., 2019). ANDSystem includes a knowledge base containing over 40 million facts about molecular-genetic interactions, including physical intermolecular interactions, gene expression regulation, activity regulation, stability, and protein transport. Work on the reconstruction and analysis of gene networks in ANDSystem is performed using the ANDVisio program. The Pathway Wizard function implemented in ANDVisio was used to reconstruct regulatory pathways, which perform search queries to the knowledge base based on a given template. A schematic description of the templates used to reconstruct regulatory pathways is provided in Supplementary Materials 1–4.


Supplementary Materials are available in the online version of the paper: https://vavilov.elpub.ru/jour/manager/files/Suppl_Demenkov_Engl_27_7.pdf


Patient- and tissue-specific gene expression and DNA methylation data. Patient-specific and tissue-specific data on gene expression and DNA methylation were used to reconstruct gene networks. Tissue-specific gene expression data was used to filter gene networks using built-in ANDSystem methods. Information on tissue-specific gene expression was represented in ANDSystem. Information on differential gene expression was taken from the GEO database (Barrett et al., 2013; https://www.ncbi.nlm.nih.gov/geo/). Experiments were selected for which results of hepatocarcinoma tissue samples obtained from patients with this disease were available. The statistical significance values of differential gene expression and differential methylation in hepatocarcinoma tumor tissue samples compared to control samples were calculated in the GEO2R software package (Barrett et al., 2013; https://www. ncbi.nlm.nih.gov/geo/geo2r/). Calculation parameters were selected by default.

FDA-approved pharmacological targets. Data on FDA-approved pharmacological targets were extracted from the Human Protein Atlas resource (Uhlén et al., 2015; https://www.proteinatlas.org/).

Potential pharmacological target prioritization method. The criteria presented in Table 1 were used to prioritize candidate genes for pharmacological targets. The resulting gene weight was assessed as the sum of the weights of all criteria.

**Table 1.**
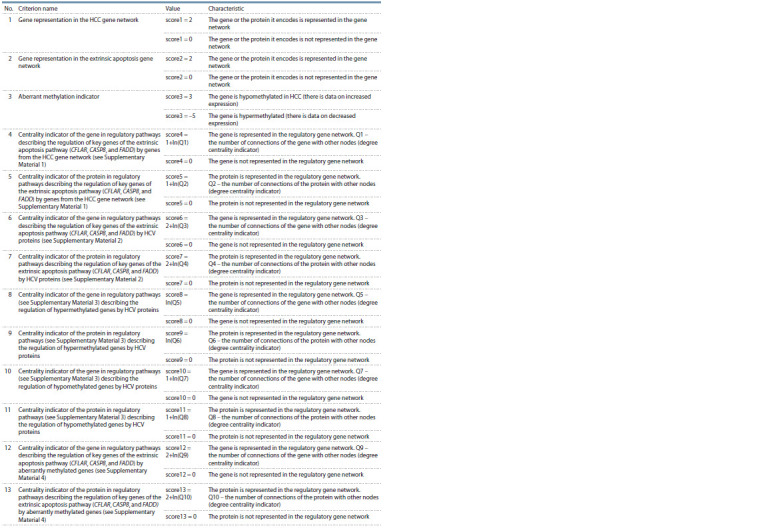
Criteria developed for prioritizing candidate genes of pharmacological targets

## Results and discussion

To prioritize potential pharmacological targets, we applied 13 criteria considering various characteristics of the structural and functional organization of liver cancer gene networks and programmed cell death, including patient- and tissue-specific data on DNA methylation. Each criterion was assigned a quantitative weight indicator. The sum of the indicators for all 13 criteria was calculated as the resulting characteristic. To rank the genes by priority, they were arranged in a list from higher to lower values of the total indicator. Thus, genes with higher priority as candidates for pharmacological targets were at the top of the list (i. e., they had a lower rank).

When calculating the weight indicators of genes by prioritization criteria, the reconstruction of the gene networks of hepatocellular carcinoma (HCC) and the extrinsic apoptosis pathway was carried out as described below.

Reconstruction of the human hepatocellular carcinoma gene network

The automated search for genes associated with HCC, conducted using the new version of ANDSystem (Ivanisenko V.A. et al., 2019), identified more than 5,100 genes. Subsequently, ANDSystem built-in methods were used to filter genes by tissue specificity, retaining only the genes expressed in the liver – 4,905 genes. A list of 1,211 differentially expressed genes (DEGs) was then used based on RNA-seq analysis from the study by Huang et al. (2011). These data were obtained from the tissues of ten patients with HBV-associated HCC. Healthy tissues from the same patients were used as controls.

Following this step, the intersection of the gene network was reconstructed with ANDSystem, and the list of differentially expressed genes was carried out using ANDVisio built-in functions. As a result of the intersection, the gene network retained 584 genes found by ANDSystem methods to be associated with hepatocellular carcinoma based on data from published works and databases, which were also present in the list of differentially expressed genes of human hepatocellular carcinoma obtained from RNA-seq data in (Huang et al., 2011). Asearch was then conducted for proteins expressed from these genes and metabolites associated with these proteins through direct interactions (a ʻcatalystʼ type association), and a network of interactions between all objects in the gene network (genes, proteins, and metabolites) was reconstructed. The gene network contained 584 genes, 580 proteins, 1,061 metabolites, and over 16,000 interactions at this stage.

The gene network was expanded in the second stage with patient- and tissue-specific DNA methylation data (Supplementary Material 5). This included 67 genes with differentially altered methylation (hyper- or hypomethylated genes) in patient tumors compared to control samples. After adding aberrantly methylated genes and their protein products and expanding the gene network with metabolites interacting with them, the final gene network contained 627 genes, 624 proteins, 1,105 metabolites, and 17,387 interactions.

Reconstruction of the extrinsic apoptosis pathway gene network

The gene network of the extrinsic apoptosis pathway was reconstructed considering GeneOntology and ANDSystem data (Supplementary Material 6). Initially, a list of genes involved in the extrinsic apoptotic signaling pathway was formed using a query to the GeneOntology database. The following keywords were used for the query: GO term “extrinsic apoptotic signaling pathway”, organism “human”. Based on this query, a list of 259 genes was obtained. This list was then uploaded into the ANDVisio program to construct a gene network. Using ANDSystem, the gene network was expanded with proteins expressed from the entered genes, as well as with metabolites associated with these genes. As a result, the gene network of the extrinsic apoptosis pathway contained 259 genes, 260 proteins, and 513 metabolites.

Gene prioritization results

A total of 1,345 genes were analyzed, including participants in the HCC and extrinsic apoptosis pathway gene networks and regulatory pathways. The results of applying prioritization criteria for the top 30 priority genes are presented in Table 2. Out of 1,345 genes, 137 were targets of FDA-approved drugs. The top 100 priority list included 19 genes targeted by FDA-approved drugs. Detailed information on the results of prioritization for the 100 highest priority genes, containing quantitative values for each of the criteria, is provided in Supplementary Material 7. Of these 19 target genes, 17 are characterized as cancer-related genes. According to the hypergeometric distribution, the probability of an event in which 17 or more out of 19 selected genes are associated with cancer is p = 0.0002. This analysis signifies that the top 100 priority genes in the table of potential targets are statistically significantly associated with cancer (significance level p = 0.0002).

**Table 2.**
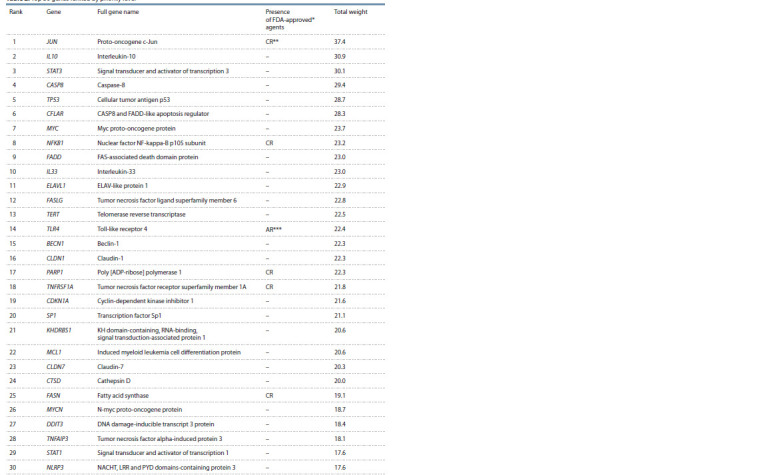
Top 30 genes ranked by priority level FDA – Food and Drug Administration, the agency of the US Department of Health and Human Services responsible for the sanitary supervision of food products and medicines; ** CR – cancer-related genes; *** AR – genes related to the “age-related macular degeneration” disease.

The calculation of prioritization criteria indicators, based on the reconstruction of regulatory pathways (criteria 4–13), was conducted automatically using ANDSystem with the templates provided in Supplementary Materials 1–4. The reconstruction and analysis of regulatory pathways of hypermethylated genes by Hepatitis C viral proteins, the results of which were used in prioritization criteria 8–11, have been previously described by us (Antropova et al., 2022).

The JUN gene occupies the top rank in the table (see Table 2). It belongs to the group of drug target genes approved by the FDA and is also associated with cancer (cancer-related genes). Numerous literature reports discuss its role in various types of cancer. For instance, it has been shown that JUN affects the development of colon cancer (Nateri et al., 2005) and that activated JUN is predominantly expressed at the invasive front of breast cancer and is associated with proliferation and angiogenesis (Vleugel et al., 2006).

**Fig. 1.**
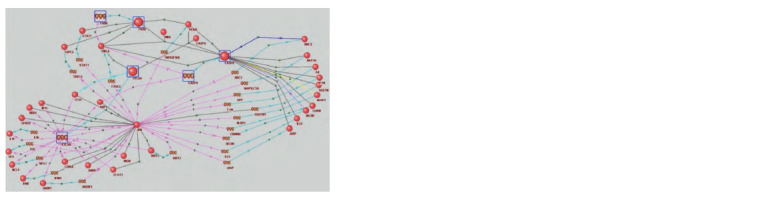
Interaction network reconstructed using ANDSystem, through which JUN can regulate key apoptosis proteins – CFLAR, CASP8, and FADD Spheres represent proteins, and spirals symbolize genes. Black lines indicate physical interaction, turquoise arrows denote expression, pink arrows signify regulation of expression, blue arrows represent transport regulation, and yellow arrows indicate activity regulation

According to our results, this gene could regulate the extrinsic apoptosis pathway. The regulatory network we reconstructed, which describes the molecular pathways through which JUN could regulate the extrinsic apoptosis pathway markers CFLAR, CASP8, and FADD, is presented in Figure 1. The regulatory network is based on various conclusions from experimental studies. For example, it has been shown that FASLG expression depends on JUN – irradiation increased FASLG expression in GCK cells via the activation of the JNK/c-Jun signaling pathway (Dong et al., 2016). The FASLG gene encodes the TNFL6 protein, a cytokine that binds to the TNFRSF6/FAS receptor, transmitting an apoptosis signal to cells. In another study (Liu Z. et al., 2019), deletion of FASLG inhibited the expression of CASP8, demonstrating another possible way for JUN to influence apoptosis (via CASP8).

It should be noted that pharmacological targets approved by the FDA, which are not associated with cancer but may be related to apoptosis, also present a particular interest. Specifically, in our table, TLR4 (ranked 14th) stands out among such genes. According to the FDA, the TLR4 gene is associated with “age-related macular degeneration” disease. Disruption of apoptosis is a key pathological factor in this disease (Yi et al., 2012).

**Fig. 2.**
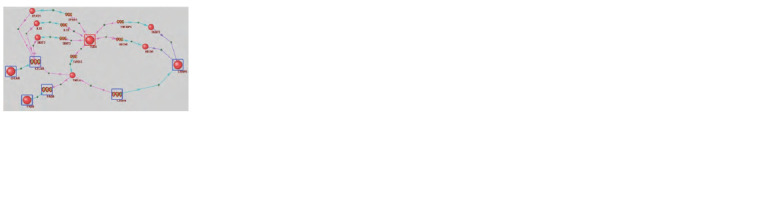
Interaction network reconstructed using ANDSystem, through which TLR4 can regulate key apoptosis proteins – CFLAR, CASP8, and FADD. Spheres represent proteins, and spirals symbolize genes. Turquoise arrows indicate expression, purple arrows represent regulation, and pink arrows denote expression regulation.

The regulatory network describing the molecular pathways through which TLR4 can regulate CFLAR, CASP8, and FADD is presented in Figure 2. For instance, one can observe the regulatory influence from TLR4 to TNFAIP3. It is reconstructed based on a published study, showing that TLR4 activates a signaling pathway leading to the activation of NF-κB transcription factor. NF-κB, in turn, induces the expression of TNFAIP3, as demonstrated in endothelial cells (Soni et al., 2018). TNFAIP3 increases the level of cleaved caspase-8, as confirmed by knockdown, while overexpression of TNFAIP3 has the opposite effect (Liu K. et al., 2018). Similarly, TLR4 could enhance the expression of Beclin-1 through NF-κB (Copetti et al., 2009), which induces caspase-8 cleavage, leading to autophagy and apoptosis (Song et al., 2014).

**Fig. 3.**
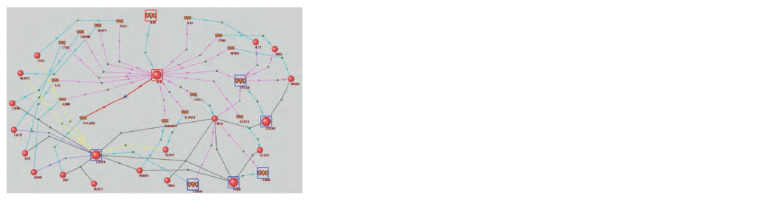
Interaction network reconstructed using ANDSystem, through which IL10 can influence CFLAR, CASP8, and FADD. Spheres represent proteins, and spirals symbolize genes. Black lines indicate physical interaction, turquoise arrows denote expression, purple arrows represent regulation, pink arrows signify regulation of expression, and yellow arrows indicate activity regulation.

The IL10 gene occupies the second rank in the table. It belongs to the group of genes not included in the list of FDA-approved pharmacological targets. However, their mechanisms of influence on the development of HCC are widely discussed in the literature. In 2020, a study (Qian et al., 2020) suggested that combining IL10 and PD-L1 inhibitors may form the basis for effective treatment. The regulatory network, describing the molecular pathways through which IL10 can regulate CFLAR, CASP8, and FADD, is presented in Figure 3.

Another group consists of genes for which the FDA does not indicate approved agents, yet the mechanism of action of some widely used drugs affects these genes or the proteins they encode. This group includes the STAT3 and MYC genes, occupying the rank table’s third and seventh positions. Asubstantial number of publications indicate that STAT3 plays a crucial role in the initiation, progression, immune suppression, and metastasis of HCC. Specific drugs affect the functioning of STAT3. For instance, F.M. Gu et al. demonstrated that the inhibition of HCC growth and metastasis by the targeted anticancer drug “sorafenib” is mediated by blocking STAT3 (Gu et al., 2011). It is also known that sorafenib induces apoptosis (Xie et al., 2012). L.Wu et al., studying the mechanism of action of quercetin (a natural flavonoid included in some dietary supplements and drugs), showed that it inhibits the progression of HCC, affecting apoptosis, migration, invasion, autophagy, via the JAK2/STAT3 signaling pathway (at least partially) (Wu et al., 2019). The action mechanism of another anticancer drug – trametinib, used for melanoma treatment, is based on inhibiting the MEK protein, part of the signaling cascade. MEK inhibition reduces the MYC protein level, which promotes cell survival, and increases the pro-apoptotic protein BIM level, suppressing HCC growth (Zhou et al., 2019).

The direct markers of the extrinsic apoptosis pathway, CASP8, and CFLAR, are ranked 4th and 6th in the rank table. The TP53 gene, the importance of which for apoptosis is well known, is positioned between them at the fifth position. Thus, it can be concluded that among the potential pharmacological targets we found, the top results of prioritization (see Table 2) include genes that are indeed drug targets – either FDAapproved or drugs aimed at other targets but affecting these genes and the proteins they encode in their action mechanisms, as well as genes that are only currently being discussed as promising targets.

**Fig. 4.**
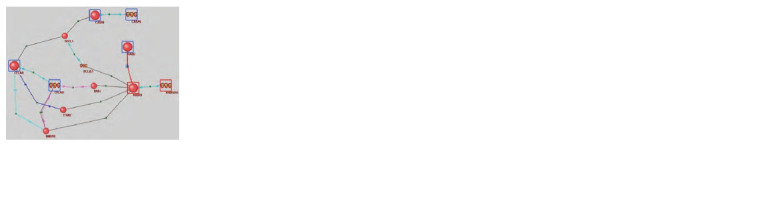
Interaction network reconstructed using ANDSystem, through which KHDRBS1 can regulate key apoptosis proteins – CFLAR, CASP8, and FADD. Spheres represent proteins, and spirals symbolize genes. Black lines indicate physical interaction, turquoise arrows denote expression, and pink arrows signify expression regulation.

Of particular interest as pharmacological targets may be genes that have been poorly studied to date in relation to HCC development mechanisms. Such genes could be fundamentally new pharmacological targets. Specifically, among such genes that made it to the top 100 highest priority list is KHDRBS1, which occupies the 21st position in the rank table (see Table 2). The regulatory network describing the molecular pathways through which KHDRBS1 can regulate CFLAR, CASP8, and FADD is presented in Figure 4.

## Conclusion

A computer reconstruction of gene networks for hepatocellular carcinoma and programmed cell death (extrinsic apoptosis pathway) was conducted, taking into account patient- and tissue-specific DNA methylation data, using the ANDSystem software and information system. Based on the 13 developed criteria, considering the specifics of the reconstructed gene networks’ structural and functional organization, potential pharmacological targets were prioritized. Six candidate genes (JUN, IL10, STAT3, MYC, TLR4, and KHDRBS1), occupying high positions in the ranked list according to prioritization results, may be of greatest interest as potential pharmacological targets.

## Conflict of interest

The authors declare no conflict of interest.
